# Analysis of motion of the rectum during preoperative intensity modulated radiation therapy for rectal cancer using cone-beam computed tomography

**DOI:** 10.1186/s13014-014-0311-6

**Published:** 2015-01-08

**Authors:** Hideomi Yamashita, Ryousuke Takenaka, Akira Sakumi, Akihiro Haga, Kuni Otomo, Keiichi Nakagawa

**Affiliations:** Department of Radiology, University of Tokyo Hospital, 7-3-1, Hongo, Bunkyo-ku Tokyo, 113-8655 Japan

**Keywords:** Rectal motion, Rectal cancer, CBCT, IMRT, SIB

## Abstract

**Purpose:**

The purpose of the present study was to quantify the inter-fractional motion of the rectum and the rectal and bladder volumes using CBCT scans taken during chemoradiation therapy (CRT) for rectal cancer. Also, assessment was made for a better margin for simultaneous integrated boost - intensity modulated radiation therapy (SIB-IMRT) for rectal cancer.

**Methods and materials:**

There were 32 patients in this study undergoing preoperative CRT for rectal cancer. Each rectum and bladder was contoured on all planning CTs and CBCTs (day 1, 7, 13, 19, 25). The target volume was configured by adding margins (0, 3, 5, 7, 10, and 15 mm) to the rectum on planning CT. The respective percentage of rectal volume that exceeds the target volume was calculated for each of these margins. The percentage of bladder volume that exceeds the bladder volume in the planning CT and motion of the center of gravity of rectum were also analyzed.

**Results:**

Planning CTs and series of each 5 CBCTs for 32 patients were analyzed in this study. The rectal volume tended to shrink week after week. The mean values (± SD) in the 32 series per patient of the percentage of rectum on the CBCTs exceeding target volume in which the margins of 0, 3, 5, 7, 10, and 15 mm were added to the rectum on planning CT were 20.7 ± 12.5%, 7.2 ± 8.3%, 3.9 ± 5.9%, 2.1 ± 3.9%, 0.7 ± 1.8%, and 0.1 ± 0.3%, respectively. No association was seen between the percentage of changes of bladder volume and motion of rectal centroid.

**Conclusions:**

In this study, we estimated the motion of the rectum using planning CT and CBCT. Ten to fifteen mm is a sufficient margin for the rectum during SIB-IMRT for rectal cancer in the supine position.

## Introduction

Neoadjuvant concurrent chemoradiation therapy (CCRT) has become the standard for locally advanced rectal cancer. Previous studies showed that preoperative CCRT reduced recurrence rate and increased sphincter preservation rate when compared to postoperative CCRT [1,2]. Intensity modulated radiation therapy (IMRT) has gradually replaced traditional four-field box radiotherapy in rectal cancer treatment because it improves dose distribution and reduces bowel exposure [3,4]. Volumetric modulated arc therapy (VMAT), a recently developed technique involving arc-IMRT delivery, has been shown to further improve dose conformity and to reduce the dose to the organ at risk [5,6].

Preoperative CRT for rectal cancer is important because local control for pelvis is closely related to cure in rectal cancer. Additionally, rectal cancer could be curable even if there are a few lung or liver metastases called as oligometastases or oligo-recurrence [7,8] after local therapy like surgery, stereotactic radiotherapy, or radiofrequency ablation [9].

It is clear that the quantification of rectal tumor and mesorectal motion is required to improve the certainty of target volume (TV) coverage. Similarly, information on normal tissue placement throughout a treatment course is necessary to estimate the true risk to these organs. Rectal and other organ motion is most critical in delivering radiation therapy (RT) when the target volume conforms more closely to the rectum and the dose reaches a more critical level. A careful assessment of the internal margins, which must be added to the TV to compensate for physiologic variations in the size, shape, and position of the TV during RT, would contribute toward optimization of the therapeutic ratio.

The purpose of this study was to quantify the rectal movement as well as the changes in rectal and bladder volumes using cone-beam computed tomography (CBCT) scans taken during CCRT for rectal cancer.

## Materials and methods

### Patients & eligibility

The patients were consecutive cases who received preoperative CRT by VMAT IMRT using a simulataneous integrated boost (SIB) for stage II-III rectal cancer with invasion to the rectum/below the peritoneal reflection (Rb) from January 2012 to August 2013 in our department. The name of the body is “analysis about organ motion during radiation therapy for body tumor”. The reference number is No. 2613. For dose fractionation, the schedule was 45 Gy in 25 fractions at 95% dose for elective volume and 55 Gy in 25 fractions at 95% dose for the boosted volume. The chemotherapy consisted of 300 mg/m^2^/day of oral tegafur/uracil (UFT) and 75 mg per day of Leucovorin (UZEL) on the same day as RT for 25 days.

The patients’ eligibility criteria included: 1) histologically confirmed rectal adenocarcinoma, 2) clinically diagnosed T3-4 or node-positive disease by use of trans-rectal ultrasound, 3) no distant metastasis, 4) no prior chemotherapy, 5) no prior RT in pelvic cavity, 6) curative aim of total mesorectal excision (TME) after CCRT, 7) no other simultaneous malignancies, 8) lower edge of primary tumor invading lower rectum (Rb), 9) over 18 years old, and 10) having PET/CT examination before preoperative therapy.

A total of 32 cases (23 males and 9 females) were included in this study. The patients’ characteristics are shown in Table 1. All cases invaded the Rb and the median distance from the anal verge (AV) was 5 cm (range: 2-12 cm). Clinical T-stages of cT2/T3/T4 consisted of 2/23/7 cases, respectively. Regarding pathological stages of pT0/T1/T2/T3/T4/Tx, there were 5/2/13/10/2 cases, respectively.

**Table 1:** **Patient and tumor characteristics**

**Characteristics**	**Number**	**Rate**
Age (y)		
Median	66	
Range	44-85	
Distance from AV		
Median	5 cm	
Range	0-12 cm	
Rectal volume on planning CT		
Median	70 cc	
Range	43-199 cc	
Bladder volume on planning CT		
Median	177 cc	
Range	44-537 cc	
Sex		
Male	23	72%
Female	9	28%
Tumor		
1/4 Circumference	3	9%
1/3 Circumference	5	16%
1/2 Circumference	15	47%
2/3 Circumference	3	9%
3/4 Circumference	1	3%
Whole Circumference	5	16%
Location		
Ra-Rb	6	19%
Rb-Ra	1	3%
Rb	17	55%
Rb-Rp	5	17%
Rb-Ra-Rp	2	6%
cT Stage		
cT2	1	3%
cT3	23	72%
cT4	7	22%
cTx	1	3%
pT Stage		
pT0	5	16%
pT1	2	6%
pT2	9	28%
pT3	10	31%
pT4	4	13%
pTx	2	6%

### Image assessment & analysis

The planning CT was acquired before RT and CBCTs were acquired once per week during RT for a total of 5 times for each patient. Rectal cancer patients were treated in the dorsal position. All of the planning CTs and CBCTs were also taken in the dorsal position. Before all of the planning CT and CBCT scans were taken, all patients were instructed to collect urine for two hours and were not regularly given laxatives to empty the intestine. All CBCT images for each week and each patient were imported into a Pinnacle3 treatment-planning workstation (Philips Healthcare, Andover, MA; ADAC, Milpitas, CA). The evaluations of organ motion and volume changes were performed on a Pinnacle3.

CT images for treatment planning were acquired using Aquillion TMLB (TOSHIBA, Tokyo, Japan). CT images were acquired with 2-mm-thick slices. The CBCT images acquired immediately before treatment were applicable for the accurate localization of the target. The pre-treatment 3D CBCT images were acquired with kV imaging parameters of a beam of 120 kVp and 20 mA per 20 ms at an axial field length of 20 cm with a bow-tie filter immediately before daily treatment. The typical number of frames was approximately 650 in a pre-treatment CBCT scan. In the registration procedure, Chamfer matching (bone matching) was used.

**Figure 1:**
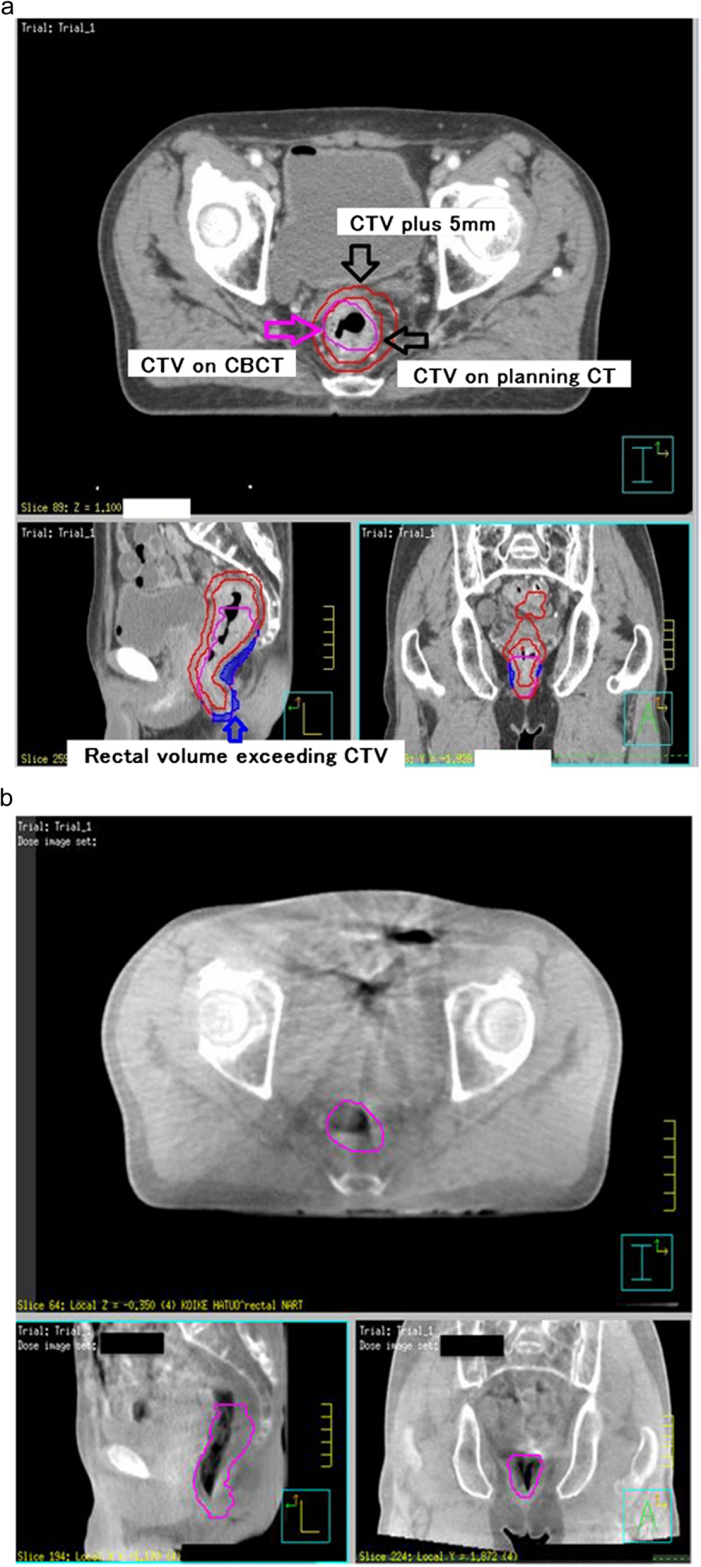
**Contouring of the rectum on planning CT (a) and CBCT on 7 days (b).** In Figure 1a, the inner red line is CTV (= rectal volume) countured on planning CT, the outside red line is CTV plus 5 mm margin, the pink line is CTV on CBCT on 7 days, and the blue mesh is CTV on CBCT exceeding CTV on planning CT plus 5 mm.

At first, each rectum and bladder was contoured on every scanned CT image. The outer wall of the rectum was contoured and the upper edge was up to one slice below the start of the sigmoid flexure and the lower edge was up to the anal verge (Figure 1). We are looking at CTV. Each rectum and bladder was contoured on all planning CTs and CBCTs (day 1, 7, 13, 19, 25). Secondly, the target volumes as PTV were calculated by adding margins of 0, 3, 5, 7, 10, and 15 mm to the CTV of the rectum on planning CT. These target volumes were compared with rectal volumes on CBCTs (32 cases × 5 times = total 160 series) and the percentage of rectal volume on CBCT that exceeded each target volume was calculated. This percentage was calculated as the rectal volume on CBCT that exceeded each rectal volume on planned CT. Both the rectum and bladder of days 0, 1, 7, 13, 19, and 25 per patient were delineated independently. Therefore, the shrinking of the volume over time can be seen.

The caudal edge of the rectum was defined as the anal verge and the rectum was contoured up to the slice wherein the anterior wall of the intestine started to shift into the ventral side of the cranial edge and, in sum, up to the sigmoid colon.

The percentages of changes of the bladder volumes and motions of the center of gravity of the rectum were also analyzed. The changing percentage was calculated as bladder volume for each CBCT per bladder volume on planned CT. The motion of the center of gravity of the rectum was calculated as the anterior-posterior, left-right, and cranio-caudal position gaps between the coordinates of the center of gravity of the rectum on each CBCT and rectal centroid on planned CT.

Student’s t test and Fisher’s exact test (one tailed) were used to test the significance of differences between cohorts. Pearson’s product-moment correlation coefficient was used to examine the relationship between the bladder volume and the motion of the rectal center of gravity.

### Consent

Written informed consent was obtained from the patient for the publication of this report and any accompanying images.

## Results

The mean rectal motion (± SD) of the center of gravity in 32 series of CT sets per patient was +5.6 (±7.3) mm in the cranio-caudal direction, -2.2 (±5.0) mm in the ventro-dorsal direction, and -0.9 (±2.6) mm in the right-left direction, respectively. The mean value (± SD) of 32 SDs in the respective 32 patients was 3.8 (±2.1) mm in the cranio-caudal direction, 2.7 (±2.8) mm in the ventro-dorsal direction, and 1.3 (±0.6) mm in the right-left direction, respectively. The maximum and minimum values of 32 SD sets were 8.3 mm and 0.8 mm in the cranio-caudal direction, 17.0 mm and 1.0 mm in the ventro-dorsal direction, and 2.5 mm and 0.2 mm in the right-left direction, respectively.

**Figure 2:**
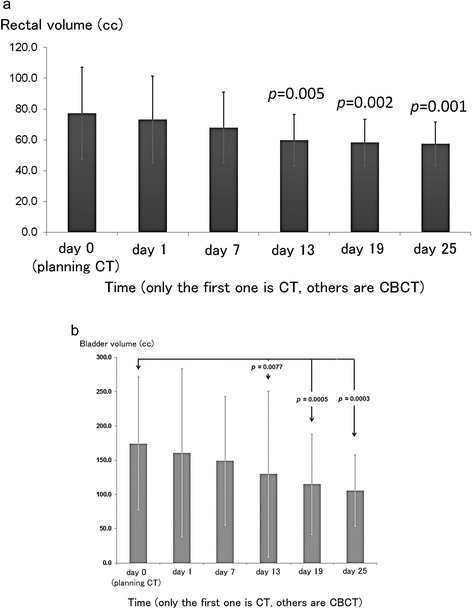
**Rectal (a) and bladder (b) volumes of planning CT and CBCT.**

Planning CTs and series of each 5 CBCTs for 32 patients were analyzed in this study. The mean rectal volumes (± SD) on planning CT and CBCTs (on days 0, 1, 7, 13, 19, and 25) were 77.3 (±29.9) cc, 73.3 (±28.2) cc, 67.9 (±22.9) cc, 59.8 (±16.7) cc, 58.2 (±15.2) cc, and 57.5 (±14.1) cc, respectively. The mean bladder volumes were 174.3 (±97.1) cc, 160.5 (±122.7) cc, 149.0 (±94.1) cc, 129.7 (±120.9), 115.0 (±73.0) cc, and 105.4 (±52.2) cc. The rectal volume tended to shrink week after week and the shrinking comparisons between planning CT versus CBCT were significant on day 13 (*p* = 0.005), day 19 (*p* = 0.002) and day 25 (*p* = 0.001) (Figure 2a) and for the bladder were significant on day 13 (*p* = 0.0077), day19 (*p* = 0.0005), and day 25 (*p* = 0.0003) (Figure 2b) by paired *t*-test.

**Figure 3:**
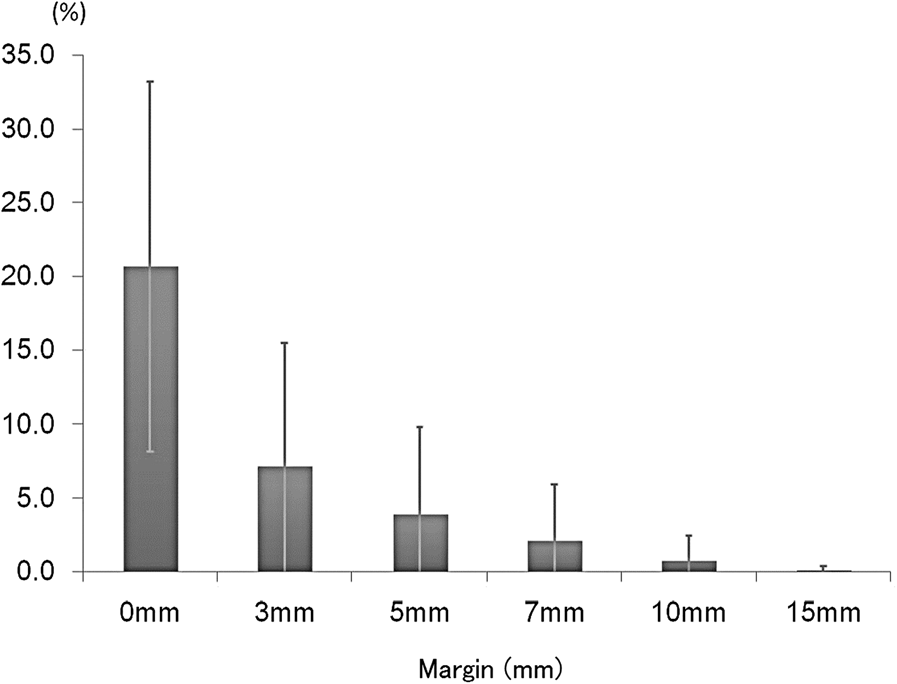
**The percentages of rectum changes on the CBCTs exceeding target volumes in which margins of 0 mm, 3 mm, 5 mm, 7 mm, 10 mm, and 15 mm were added.**

The mean values (± SD) in the 32 series of the percentages of the rectum exceeding target volumes on CBCTs per patient in which the margins of 0 mm, 3 mm, 5 mm, 7 mm, 10 mm, and 15 mm were added to the rectum on planning CT were 20.7 ± 12.5% (range 3.3-61.1%), 7.2 ± 8.3% (range 0.1-40.0%), 3.9 ± 5.9% (range 0-30.4%), 2.1 ± 3.9% (range 0-21.3%), 0.7 ± 1.8% (range 0-10.3%), and 0.1 ± 0.3% (range 0-2.7%), respectively (Figure 3).

**Figure 4:**
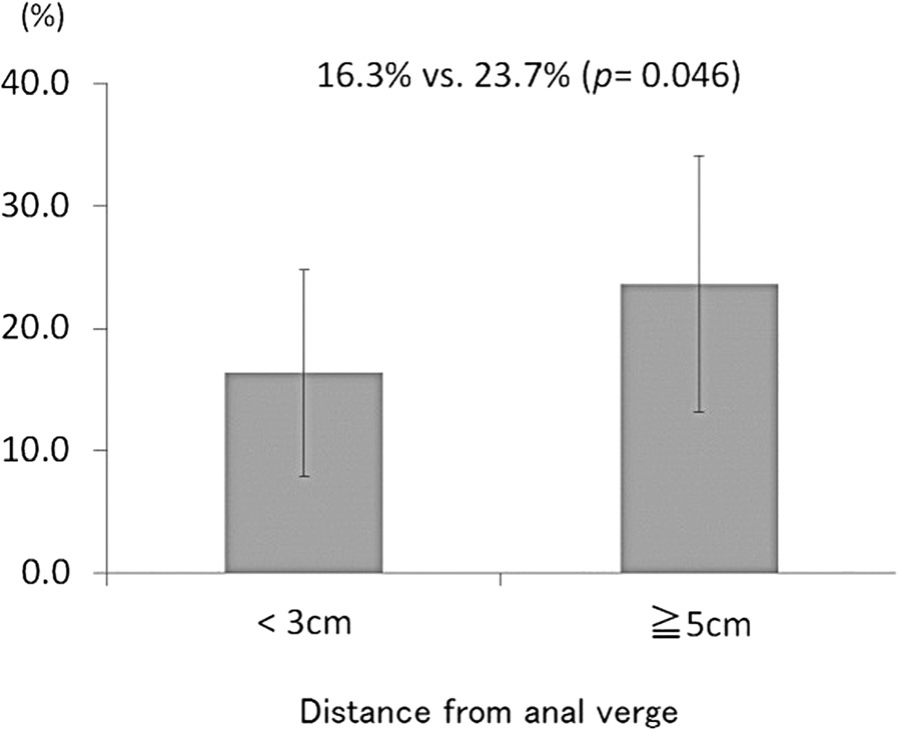
**The percentages of rectal volume changes exceeding target volumes in which margins of less than 3 cm, were compared with those over 5 cm on the distance from AV.**

The associations between sex, clinical stage, and the distance from AV versus the change of the rectal volume were also analyzed. The percentages of rectal volume exceeding target volume in which the margin was 0 mm were compared between male vs. female, cT2/3 vs. cT4, and less than 3 cm vs. over 5 cm on the distance from AV. The values were 19.8% vs. 22.8% (*p* = 0.48), 22.5% vs. 14.3% (*p* = 0.06), and 16.3% vs. 23.7% (*p* = 0.046), respectively (Figure 4). In regard to the distance from AV, when the tumor location was close to the AV, the excessive rectal volume was significantly smaller.

**Figure 5:**
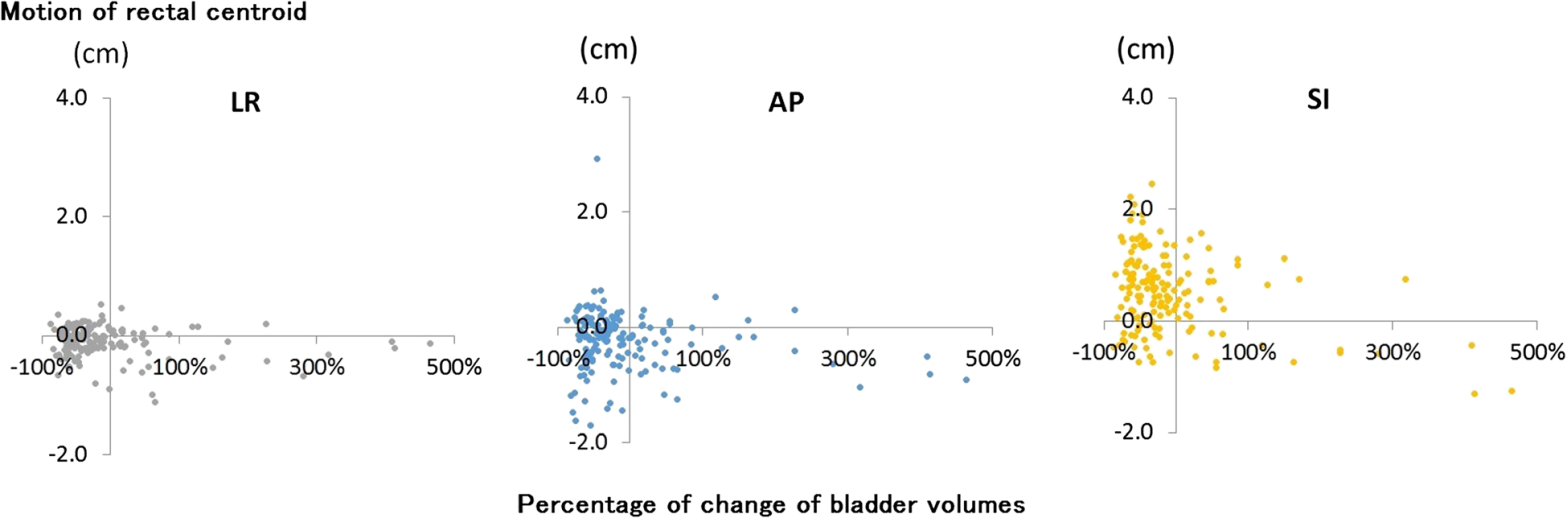
**The correlation between the percentage of change of bladder volumes (horizontal axis) and motion of rectal centroid (vertical axis).** The percentage of change of bladder volumes was calculated as {(bladder volume in CBCT) – (bladder volume in planning CT)} / (bladder volume in planning CT) × 100 (%).

The percentage of bladder volume changes and motion of rectal centroid were analyzed in order to check whether the rectal motion depends on the bladder volume. The coefficient of correlation for the percentage changes of bladder volume and motion of rectal centroid in the left-right direction was R = -0.10, R = -0.10 in the anterio-posterior direction, and R = -0.27 in the cranio-caudal direction. No significant association was seen in all directions (Figure 5).

## Discussion

The purpose of the present study was to quantify the motion of the rectum using CBCT during neoadjuvant CCRT for rectal cancer, and to consider a better margin for SIB-IMRT for rectal cancer. This study addressed positional and volumetric changes in rectal and bladder volumes in patients receiving CRT for rectal cancer. The work is original with respect to rectal cancer and patient positioning during CRT. Several studies have reported on rectal motion based on CBCT [10,11]. However, to the best of our knowledge, this is the first study to evaluate the percentage of rectum motion on the CBCTs exceeding target volume in planning CT during neoadjuvant CRT for rectal cancer.

The tendency for a reduction of the rectal volume during the weekly treatment period was seen in this study. Some studies, including our present one, have seen a decrease in the rectal volume [12,13]. As for the cause of the decreases in rectal volume in this study, the reduction of rectal tumor and the tendency for the treatment to inhibit stool formation were considered. However, it was pointed out that the decrease of the rectal volume occurs even when examining rectal change during RT for prostate cancer. The possibility remains that other factors influence the decreases of rectal volume.

In addition, in regard to the percentage of rectal volume exceeding the planning CT on CBCT, those cases with tumor less than 3 cm from the AV had significantly smaller percentages than cases with more than 5 cm. In pursuing the question of the influence of inter-fraction motion, Chong *et al*. [10] analyzed the motion by dividing the rectum into upper, middle, and lower parts and found that rectal motion was smallest in the lower part. Our results concurred with these observations.

In this analysis the change of bladder volume did not correlate with the motion of the rectal center of gravity. That the patients were irradiated in a urine collection state for 2 hours before treatment in our institution may be a factor, and resulted in a smaller percentage of change of the daily bladder volume.

After analyzing how much of the rectum during RT is covered by the margins added to planning CT in this study, the percentages found to protrude were 0.7% +/-1.8% in a 10 mm margin and 0.1% +/- 0.3% in a 15 mm margin. Judging from this result, the margin around 10-15 mm to the rectum was considered sufficient. This result was similar to previous reports of 14.2-17 mm for the anterior wall and 14.4-16 mm for the posterior wall [10].

However, in this study, motions of the rectum in the anterior, posterior, right and left directions were not analyzed. In addition, the past studies reported were of rectal motion in patients treated in the prone position, [10,11,14,15] whereas the rectal cancer patients in our institution were treated in the supine position. The optimal margin may change with posture during irradiation or motion directions of the rectal wall, and further studies will be necessary.

Although CBCT was used for analysis in this study, the image quality of CBCT scans were generally poor and easily affected by the internal gas. Further improvement of image quality in future CBCT studies will be expected to rigorously compare contours of CBCT images.

Our clinical result of pathological response rate was 60% at surgery of total mesorectal excision. The extended margins may have compensated for the movements and changes in volume of target organs. This will also need to be confirmed in future studies.

## Conclusion

In this study, the motion of the rectum was estimated using planning CT and CBCT. Ten to fifteen mm is sufficient as a margin to the rectum during SIB-IMRT for rectal cancer in the supine position. However, the margin estimated in this study was calculated from the percentage of rectum motion on the CBCTs exceeding the planning CT, and the margin to each direction of the rectal wall was not examined. In the future, further detailed investigation is required on the difference in rectal motion according to the location of the rectal tumor above the AV. It is also necessary to examine individual margins to anterior-posterior and lateral directions.
